# Supplementary material for: Targeting Non-Alcoholic Fatty Liver Disease with Hawthorn Ethanol Extract (HEE): A Comprehensive Examination of Hepatic Lipid Reduction and Gut Microbiota Modulation
Source: Nutrients. 2024 Apr 29;16(9):1335. doi: 10.3390/nu16091335 (PMC11085873; doi:10.3390/nu16091335)

Targeting Non-Alcoholic Fatty Liver Disease with Hawthorn Ethanol Extract (HEE): A Comprehensive Examination of Hepatic Lipid Reduction and Gut Microbiota Modulation

Tianyu Wang1, Dawei Wang2, Yinghui Ding1, He Xu1, Yue Sun1, Jumin Hou1, Yanrong Zhang2*

1 → College of Food Science and Engineering, Changchun University; Changchun 130022, China. wangty84@ccu.edu.cn (T.W.); 220302116@ccu.edu.cn(Y.D.); 220302110@ccu.edu.cn(H.X.); 220301083@ccu.edu.cn(Y.S.); houjm@ccu.edu.cn(J.H.)

2 → School of Food Science and Engineering, Jilin Agricultural University; Changchun 130118, China. wangdawei@jlau.edu.cn(D.W.)

***Corresponding author.**

E-mail address: zhangyanrong@jlau.edu.cn(Y.Z).

Table S1. Composition of diets*

Ingredient (g/kg)	HFD (TP 23300)	LFD (TP 23302)
Casein	259	189
Maltodextrin	166	108
Sucrose	91	67
Corn starch	0	497
Soybean oil	33	21
Lard	313	21
Cellulose	67	48
Mineral mix (M1020)	52	36
Vitamin mix (V1010)	13	9
L-cystine	3	2
Choline bitartrate	3	2
Tertiary butylhydroquinone (TBHQ)	0.067	0.067
Total	1000.067	1000.067

*Diets were purchased from TROPHIC Animal Feed High-tech Co., Ltd. (Nantong, China), and information on diet composition was provided by the company.

Table S2. Primer sequences used for real-time quantitative PCR

Gene	Forward (5'-3')	Reverse (5'-3')	PrimerBank ID
FASN	GGAGGTGGTGATAGCCGGTAT	TGGGTAATCCATAGAGCCCAG	30911099a1
MGL	CGGACTTCCAAGTTTTTGTCAGA	GCAGCCACTAGGATGGAGATG	6754690a1
HSL	CCAGCCTGAGGGCTTACTG	CTCCATTGACTGTGACATCTCG	26325924a1
ATGL	GGATGGCGGCATTTCAGACA	CAAAGGGTTGGGTTGGTTCAG	26327465a1
GAPDH	AGGTCGGTGTGAACGGATTTG	TGTAGACCATGTAGTTGAGGTCA	6679937a1

Figure S1 Heatmap of cluster analysis for each asample

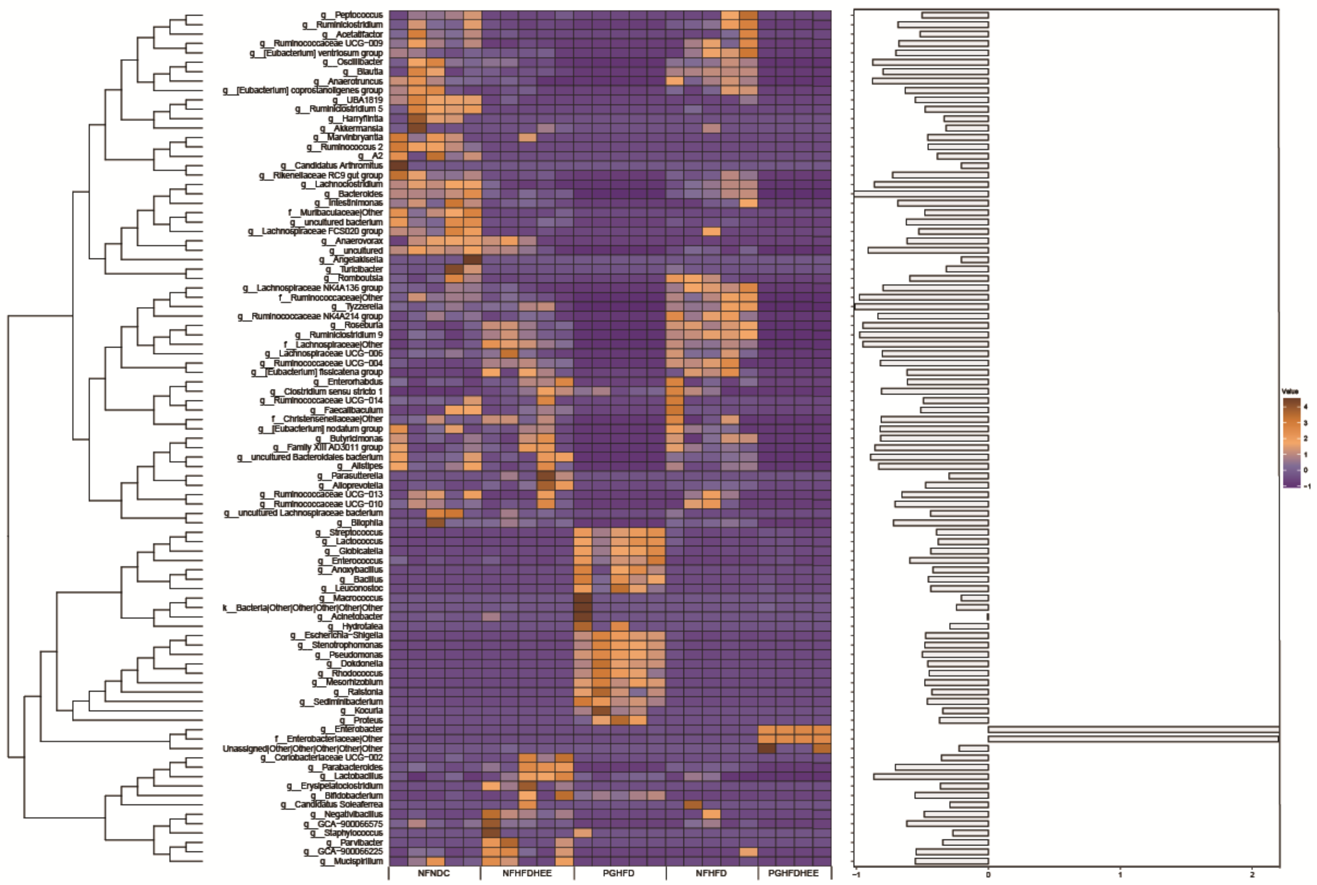

Supplement: Supplementary file 1 [file nutrients-16-01335-s001.zip › nutrients-2930682-supplementary.pdf]
